# Sensory Perception and Physicochemical Characteristics of Geisha Coffee From Different Production Zones in Panama

**DOI:** 10.1002/fsn3.71278

**Published:** 2025-11-25

**Authors:** Diana Batista Ledezma, Camilla Sartori, Elizabeth Tomasino

**Affiliations:** ^1^ Department of Food Science & Technology Oregon State University Corvallis Oregon USA

**Keywords:** consumers, multiple factor analysis, place‐of‐origin, RATA, terroir

## Abstract

Coffee *terroir* highlights distinct characteristics shaped by its origin, giving it identity and added value that is especially appealing to consumers in specialty markets. Factors including environmental conditions, farming practices, and processing techniques are important in defining a product's origin. Unique characteristics from the previous factors have positioned Panama favorably in the specialty coffee market. Specifically, the Geisha coffee variety is Panama's most valuable coffee, but there is limited information on Panama's coffee *terroir*. This study aimed to determine whether there are distinct *terroir* qualities and if physicochemical or sensory attributes differentiate Geisha coffees across Panama production zones. Green coffee beans from the 2023–2024 harvest, all washed processed, were collected from four Panama production zones: Boquete, Potrerillos Arriba, Renacimiento, and Sensory Perception. Physicochemical analyses were applied to green beans, roasted beans, and brewed coffee using standard methods. Sensory attributes such as aroma, flavor, and taste intensity were evaluated by a screened panel using the Rate‐All‐That‐Apply (RATA) method. Variables were statistically analyzed using correspondence analysis (CA), multiple factor analysis (MFA), agglomerative hierarchical clustering (AHC), and discriminant analysis (DA). Physicochemical characteristics did not drive differences across production zones. Discriminant analysis demonstrated sensory attribute differences among the production zones. The findings from this study indicated specific characteristics that reflect regional *terroir* across Panamanian coffee production zones, providing evidence that terroir qualities do exist. Our results provided valuable initial information on the uniqueness of Panama Geisha coffees from these regions and an initial collection of sensory descriptors associated with these coffees by coffee consumers.

## Graphical abstract

No significant differences based on main physicochemical parameters. Significant aroma/flavor differences of coffee Panama geisha coffee from Boquete, Tierras Altas and Renacimiento were found. Sensory differences support the existence of regional terroir.

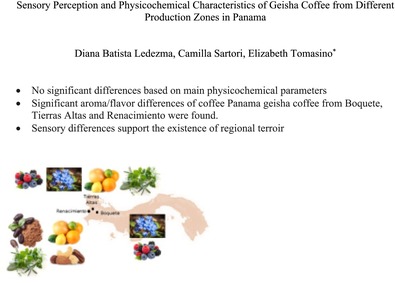

## 1 Introduction

Single‐origin coffees are highly valued for their unique sensory attributes, traceability, and sustainable production (Teuber 2010). From seed to cup, place of origin related factors come together to make each sip a distinct experience. The complex interactions between geographical, environmental, and human factors contribute to the concept of *terroir* (Charters et al. 2017). Though, commonly applied in the wine industry (Bonfante and Brillante 2022; Souza Gonzaga et al. 2021), *terroir* has also become an important aspect to understand the differentiation of other food products, including coffee (Charters et al. 2017; Silva et al. 2018; Teuber 2010). Therefore, *terroir* can highlight the characteristics that coffee develops through the specific conditions of its production of origin that make them unique.

Coffee is one of the most consumed beverages in the world. In 2022, its economic impact in the United States was estimated at approximately $343.2 billion (National Coffee Association n.d.). As of 2024, the U.S. specialty coffee retail market value was $48 billion, driven by consumers' age, income, and evolution of preferences and perception (Grand View Research n.d.; SCA 2024a). A continued positive growth for the specialty coffee market value is expected in the U.S. over the next years (Grand View Research n.d.).

The introduction of single‐origin coffee beans in the U.S. market began during what is known as the “Second Wave Coffee Movement” (Boaventura et al. 2018). Then, in the early 2000s, the “Third Wave Coffee Movement” brought a more holistic approach to experiencing coffee (Garcia et al. 2024), emphasizing coffee quality, and traceability throughout the supply chain and recognizing the relevance of coffee producers, importers, roasters, and baristas in delivering the product (Boaventura et al. 2018; Garcia et al. 2024). An important aspect of this experience was communicating relevant product information to the final coffee consumers (Garcia et al. 2024). Specialty coffee's global expansion reflects not only economic growth but also a broader understanding of coffee value, where consumers are willing to pay more for the specialty coffee experience (Pereira et al. 2021; Teuber 2010), as well as to be informed about the origin, processing methods, and brewing preparation. Essentially, consumers want to know where their coffee comes from and what makes it special.

Since the discovery of wild coffee trees in the African equatorial forests (R. F. Smith 1985), and coffee's first cultivation in the Yemen region (Ferreira et al. 2019), the popularity and demand for coffee beans increased rapidly. Coffee seeds were traded and gradually spread to different parts of the world (World Coffee Research 2024b), with coffee grown in more than 80 countries (Widjaya and Yanuarti 2024). In particular, tropical, subtropical and equatorial regions, provided the climatic factors and optimal growing conditions to cultivate coffee trees (Pereira et al. 2021). Through time, there have been two economically relevant coffee species: *Coffea canephora* var. Robusta and *Coffea arabica* var. Arabica (Ferreira et al. 2019); the latter being the most widely produced with an estimated 102.2 million bags (60 kg) for the 2023–2024 crop year (ICO 2025) and valued for its superior sensorial qualities (Barbosa et al. 2019; Decazy et al. 2003; Santamaría et al. 2023).

As with wine, coffee processing impacts the final characteristics of the beverage affecting the expression of *terroir* (Souza Gonzaga et al. 2021). Outside of growing coffee, its production is complex, with many post‐harvest processing methods, and storage parameters that are important to achieve high‐quality green coffee beans (Pereira et al. 2021). In general, three main conventional processing methods are performed: natural, honey or washed, showing clear differences in the final drink.

The main difference between natural and washed coffee is the removal of the outer layers of the coffee cherries. Honey coffees are depulped, but the mucilage remains on the beans during its drying (Williams et al. 2022). For the natural process, coffee beans are dried with the outer layer intact, whereas for washed coffee this outer layer is removed prior to fermentation, drying and finally to be stored until further roasting (Pereira et al. 2021). Interestingly, post‐harvest processing in coffee is considered part of the *terroir*, as environmental factors can determine the processing method chosen (Williams et al. 2022). During roasting, chemical precursors present in the green beans can develop more than 900 new compounds (Condelli et al. 2022; Seninde and Chambers 2020), which are responsible for distinctive sensorial profiles.

Panama has been recognized for its high‐quality coffee in specialty coffee markets, thanks to years of organoleptic coffee quality assessments and promotion efforts by the Specialty Coffee Association of Panama (SCAP). Panama Geisha, an exotic arabica variety originally from Ethiopia, is recognized for possessing its own fingerprint and exhibiting delicate floral, citrus, and tea‐like notes (World Coffee Research 2024a). The rediscovery of this coffee variety in Panama dates back to 2004, during an international coffee competition known as the “Best of Panama” organized by SCAP; the Geisha coffee stood out for its unique attributes (Hacienda La Esmeralda 2022; SCAP 2024). Now, more than 20 years later, this coffee continues to gain international attention. Just last year, a micro‐lot was auctioned for $10,013 USD per kilogram (SCAP 2024).

Even though Panama leads Geisha's higher prices in the market, its production is challenging. Its uniqueness is attributed to Panama's terroir, including a unique geographical position with diverse microclimates, rich volcanic soils, biodiversity, and high elevations (SCAP n.d.). Understanding how these factors contribute to coffee's distinctive sensorial profiles has been approached previously in other coffee production regions such as Costa Rica, Honduras, and Brazil (Avelino et al. 2005; Decazy et al. 2003; Scholz et al. 2018; J. Smith 2018). While there are some advances in understanding the composition, quality, and other aspects influencing the characteristics of Panamanian Geisha coffee (Koyner 2024; Santamaría et al. 2023; Vega et al. 2025), research remains limited. There remains a lack of sensorially driven studies across harvests that can determine the existence of *terroir* or identify attributes that differentiate Geisha coffee across regions within Panama. Although, it can take many years to define terroir, it is possible to determine if it exists with more limited data sets.

The aim of this study was to determine sensorially whether terroir differences exist within Panama using coffee beans from the 2023–2024 harvest year. To achieve this objective, coffee samples from four different production zones (Boquete, Potrerillos Arriba, Renacimiento, and Tierras Altas) were collected, roasted, and brewed under standardized conditions. A consumer panel evaluated the coffees using a list of descriptors, which were selected and rated according to the perceived intensity. Conventional physicochemical analyses were performed on green coffee beans, roasted coffee beans and brewed coffee. This study is the first to apply the terroir concept to Panamanian coffee, providing a holistic view of how terroir influences flavor. The use of standardized roasting, brewing and sensory analysis ensures that observed differences are attributed to terroir rather than other processing variations.

## 2 Materials and Methods

### 2.1 Production Zones and Coffee Samples

Coffee samples were sourced from four known coffee growing regions: Boquete, Potrerillos Arriba, Renacimiento, and Tierras Altas. These areas are recognized as important specialty coffee production zones in the western region of the Republic of Panama (SCAP n.d.). The elevation range of the production farms was from 1300 m.a.s.l. (meters above sea level) to a maximum altitude of 2000 m.a.s.l. (Table 1).

**TABLE 1:** Production zones of farms included in this study.

Production zone	No. of samples	Farms range of elevation (m.a.s.l)
Boquete	11	1450–2000
Potrerillos Arriba	2	1600–1800
Renacimiento	7	1400–1900
Tierras Altas	4	1300–1850

Green Geisha coffee beans from the 2023 to 2024 harvest were used in this study. Each sample consisted of 3.5 kg of green coffee beans processed under the washed method, dried until they reached a moisture content below 12% and packed in various vacuum‐sealed plastic bags. The coffee beans were produced, processed and selected according to the techniques and internal parameters of each farm. All the samples were shipped from Panama to Oregon State University (OSU) (Corvallis, OR, USA), where sensory and physicochemical analyses were performed. Samples were vacuum packaged and stored in dark containers (Jamison Mark IV, Maryland, US) at −21°C in a freezer to minimize the coffee beans' physical deterioration once they reached OSU.

### 2.2 Roasting Process

Roasting was performed using a Stronghold coffee roaster (S7 PRO, Stronghold Technology Inc., Odessa, Texas, USA). Preliminary roasting tests were conducted to assess the roasting temperature and time parameters to use for this study (Data not included). A cleaning and preheating process was completed on the roaster before samples were roasted to ensure consistent roasting across all samples. Based on previous research, achieving a light to medium roasting degree was our goal as at this level it is possible to preserve coffee's original traits (Sunarharum et al. 2014; Williams et al. 2022). From each sample of green coffee beans two batches of 600 g were roasted using identical roasting parameters. The roasting settings consisted of controlled internal temperature, airflow, halogen light, and bean agitation. The roasting process lasted around 8 min and was considered complete after the coffee beans reached the first crack (Figure S1), a development time of approximately 1 min and 15 s, and a Development Time Ratio (DTR) average of 15%. Coffee batches were cooled and blended in the roaster cooling tray for about 6 min. After the roasted coffee beans reached room temperature, the samples were weighed and then heat‐sealed in three‐layered food grade bags made of BOPP (Biaxially Oriented Polypropylene), PET (Polyethylene Terephthalate) aluminum foil, and Polyethylene (PE) with CO_2_ degassing valves (ODDIER, China). Each bag was labeled with a blinding code for the corresponding sensory analysis.

The coffee batches rested to de‐gas for at least 24 h at room temperature (~22°C–25°C) prior to sensory analysis.

### 2.3 Physicochemical Analysis

Conventional physicochemical analyses were performed on green and roasted beans in triplicate and the average values and standard deviations were reported.

#### 2.3.1 Green and Roasted Coffee Beans

##### 2.3.1.1 Moisture

The moisture content of green coffee beans was measured using a digital coffee moisture analyzer (COFFEE PRO MOISTURE‐MAC, Coffee Laboratory, Whitestone, VA, USA). Each sample consisted of 25 g of coffee beans.

##### 2.3.1.2 Apparent Density

The apparent density of green coffee and roasted bean samples was determined by adding the beans to a 1000 mL graduated cylinder that had previously been weighed. The green coffee beans' weight was registered and divided by the volume reporting grams per liter (g/L).

##### 2.3.1.3 Color

L*a*b* color measurements were performed in green coffee beans and roasted whole coffee beans, using a colorimeter (Chroma Meter Model CR‐410, Konica Minolta Inc., Ramsey, NJ, USA). For green coffee beans, 50 g of sample was weighed into a transparent petri dish using a white tile as a background. For roasted coffee beans 25 g of each sample was used. L* (Lightness), a* (red‐green value), and b* (yellow‐blue value) values were reported. The color values were reported using the C.I.E.L*a*b* scale. The instrument was calibrated following its manual instructions and using a calibration plate, verifying its corresponding values (D65 Y: 85.8, x: 0.3185, y: 0.3356) before initiating the measurements.

##### 2.3.1.4 Roast Degree

The roasting degree was determined by infrared analysis, using 20 g of coffee beans at room temperature (~22°C–25°C), ground to a medium coarse and using a coffee roast analyzer (KN‐201, Dipper, Bangkok, Taiwan). Roasting levels were based on the Agtron Gourmet scale from 0 to 133 range, where a high number suggests a lighter roast (Kim et al. 2022; Staub 1995).

#### 2.3.2 Brewed Coffee

To determine the °Brix, pH and titratable acidity of the brewed coffees, sample preparation.

Ground coffee samples were brewed using a ratio of 1:16 (International Standard Organization 2008), which corresponded to 37 g of fresh coffee and 592 mL of commercial distilled water. The water was heated to boiling temperature (99°C ± 1°C) and poured directly into the ground coffee. An immersion brewing method was used, utilizing a 24 oz. stainless‐steel French press (MAGICAFÉ, China). The coffee was left to steep for 4 min before pressing down the metal mesh plunger to separate the grounds from the brewed coffee. The coffees were immediately poured into thermal insulated stainless‐steel flasks (Fellow, 16 oz., Carter slide mugs, USA). The total brewed coffee resulted in 150 mL due to water absorption of the ground coffee. Once the samples cooled below 40°C, they were poured into 50 mL conical tubes. Samples were then centrifuged (Allegra X‐22 Centrifuge; Beckman‐Coulter Inc., Ramsey, MN, USA) at 2576 rcf for 10 min with an acceleration setting of 9. Measurements were done by four replicates and values were reported.

##### 2.3.2.1 °Brix

°Brix measurements of brewed coffee were taken using a portable densimeter and concentration meter (DMA 35; Anton Paar GmbH, Graz, Austria), using 2 mL of brewed coffee at room temperature (20°C ± 1°C).

##### 2.3.2.2 pH and Titratable Acidity

For pH and titratable acidity analyses, 50 mL of brewed coffee samples at room temperature (20° ± 1°C) were used. The pH was measured using a pH meter (SevenMulti, METTLER TOLEDO, Columbus, OH, USA). Titratable acidity was analyzed by titration method to pH 8.2 (HI3205EN), using a NaOH 0.1 N solution (Anokye‐Bempah et al. 2024; Batali et al. 2021).

### 2.4 Sensory Analysis

#### 2.4.1 Brewing and Sample Preparation

The roasted coffee beans were ground using a medium‐coarse grind setting 14 (~500–1000 μm, Guatemala grinder, Mahlkönig, Zurich, Switzerland), no more than 30 min before brewing. In between samples, the grinder was cleaned using ~5 g of the next coffee sample. The coffee used to “clean” the grinder was disposed of and not used for analysis.

Ground coffee samples were brewed as described in section 2.3.2. Approximately 55 mL of brewed sample was poured into espresso cups (4 oz., double wall, Sweese, Jonesborough, TN, USA) 5 min before each session. Samples were served at ~74°C ± 1°C (Aquatuff 351; ATKINS). A warmup coffee (a blend of arabica‐light roast) was prepared using a different coffee under the same brewing parameters and served to panelists to balance their palate prior to the evaluation of the samples for this study (Gotow et al. 2018; Plemmons and Resurreccion 1998).

#### 2.4.2 Participants

A total of 24 coffee consumers (nine male and 15 female), were recruited from the OSU coffee sensory database, using RedJade sensory software (RedJade Sensory Solutions LLC, Pleasant Hill, CA). All participants met the following criteria: consume coffee beverages (hot drip coffee, espresso, or americano) at least once a week, 18 years or older, non‐smokers, no taste deficits or other oral disorders, no oral lesions, not pregnant or lactating, no coffee allergies, and not taking any prescription medication that might interact with coffee/caffeine. The OSU Institutional Review Board authorized the use of human participants to conduct the study, and all participants provided informed consent prior to participation (IRB #: HE‐2024‐887). Sensory sessions were held in August 2024 at Oregon State University (Corvallis, OR), using individual white booths, with artificial light, and at room temperature between 20°C and 22°C. Each of the 24 coffee samples was evaluated in triplicate and testing was completed over 12 sessions (12 days).

#### 2.4.3 Sensory Evaluation

Descriptive analysis was conducted using a rate‐all‐that‐apply (RATA) procedure. Each session started with the warm‐up coffee to adjust the panelists' palates and mitigate first position effects (Dorado et al. 2016). Panelists then evaluated six coffee samples, presented in random order, one sample at a time, and labeled with a three‐digit blind code. The coffee serving order was based on a partially balanced complete block design.

For each sample, panelists selected which descriptors they felt described the sample and rated the intensity of selected descriptors. Panelists were instructed to first smell the sample and select/rate the descriptors (*n* = 12, Table 2). Then, they were instructed to taste the sample and select/rate the flavor and taste descriptors (*n* = 16, Table 2). Descriptors were selected according to Geisha coffee references and the coffee sensory lexicon (Koyner 2024; World Coffee Research 2017). For both aroma and flavor/taste, two “other” options were provided for panelists to provide and rate unlisted attributes. Between samples, panelists had a forced 1‐min break to cleanse their palate with provided water.

**TABLE 2:** Attributes evaluated and their corresponding descriptors.

Attribute	Aroma “A”	Flavor “F”	Taste “T”
Descriptors	Fruit	Fruit	Sweet
	Floral	Floral	Sour
	Citrus	Citrus	Bitter
	Butter	Butter	Tart
	Vanilla	Vanilla	
	Cocoa	Cocoa	
	Musty	Musty	
	Nutty	Nutty	
	Herbal	Herbal	
	Fermented	Fermented	
	Dried Fruit	Dried Fruit	
	Bergamot	Bergamot	

### 2.5 Data and Statistical Analysis

All data collected from the chemical analysis were presented as the averages of replicates across all samples from each region with their respective standard deviation. Statistical analyses were performed using Analysis of Variance (ANOVA) (α = 0.05), followed by Tukey's Honest Significance Difference (HSD) post hoc test using R Software version 4.4.1 (RStudio 2024.12.1, Posit Software, PBC formerly RStudio, PBC).

Cochran's Q test was used to determine differences in the frequency of terms used for RATA. Correspondence Analysis (CA) and Multiple Factorial Analysis (MFA) were used to identify possible correlations between variables. Agglomerative Hierarchical Clustering (AHC) and Discriminant Analysis (DA) functions were used, using the grouping factor of coffee region to observe possible group differentiations. These analyses were performed using XLSTAT Sensory (2022.5.1.1384 Lumivero, Denver CO, USA).

## 3 Results

### 3.1 Physicochemical Characteristics

The general physicochemical analyses of green coffee beans, roasted coffee beans, and brewed coffee are grouped by their respective production zones and presented in Table 3.

**TABLE 3:** Analysis of variance of physicochemical characteristics of green coffee beans, roasted coffee beans and brewed coffee by production zone.

Category	Production zone	Moisture (%)	Apparent density^a^ (g/L)	Roast degree^b^‐Agtron Gourmet	L*^a^	a*^a^	b*^a^	°Brix	pH	Titratable acidity (mL NaOH)
Green coffee beans	B	9.82 ± 0.75^a^	716.69 ± 10.81^a^	—	46.82 ± 1.50	2.32 ± 0.41	13.84 ± 1.07^ab^	—	—	—
	P	8.68 ± 0.79^b^	715.77 ± 17.60^ab^	—	47.17 ± 1.98	2.21 ± 0.39	14.41 ± 0.15^ab^	—	—	—
	T	9.60 ± 0.44^a^	696.34 ± 4.36^c^	—	47.95 ± 1.58	2.10 ± 0.18	14.31 ± 1.66^a^	—	—	—
	R	9.93 ± 0.28^a^	709.23 ± 6.40^b^	—	47.26 ± 1.97	2.09 ± 0.43	12.99 ± 1.10^b^	—	—	—
Roasted coffee beans	B	—	378.05 ± 13.48	68.85 ± 1.96	27.17 ± 1.30	6.65 ± 0.30	7.37 ± 0.62	—	—	—
	P	—	378.83 ± 2.95	67.60 ± 2.32	26.24 ± 1.70	6.63 ± 0.43	7.15 ± 0.78	—	—	—
	T	—	385.12 ± 19.66	67.91 ± 1.54	26.40 ± 1.08	6.51 ± 0.38	7.54 ± 0.65	—	—	—
	R	—	374.63 ± 20.42	67.81 ± 1.40	26.39 ± 0.86	6.72 ± 0.37	7.92 ± 0.55	—	—	—
Brewed Coffee	B	—	—	—	—	—	—	1.45 ± 0.08^a^	4.91 ± 0.07	6.20 ± 0.34^a^
	P	—	—	—	—	—	—	1.35 ± 0.05^b^	4.85 ± 0.05	6.35 ± 0.18^a^
	T	—	—	—	—	—	—	1.38 ± 0.04^b^	4.86 ± 0.04	6.19 ± 0.31^ab^
	R	—	—	—	—	—	—	1.38 ± 0.05^b^	4.90 ± 0.12	5.95 ± 0.44^b^

*Note:* Data are expressed as mean ± standard deviation. Different letter superscripts (^a,b,c^) in the same column indicate statistical differences (*p* < 0.05), following pairwise comparison by Tukey's HSD test.

^a^ Whole beans.

^b^ Ground coffee.

#### 3.1.1 Green Coffee Beans

Samples from Potrerillos Arriba (P) were found to have a lower moisture content than the other production zones. The density was different across production zones, with T having the lowest density followed by R. B and P density were not significantly different. Color values (L*, a*, b*) for whole beans were also similar across regions in terms of lightness, red‐green, and yellow‐blue color variation.

#### 3.1.2 Roasted Coffee Beans

After the roasting process, coffee beans did not show a significant difference in density, roasting degree (Agtron), and color values across their respective production zones. As expected, the density was reduced after roasting, which is generally attributed to moisture loss during this process.

#### 3.1.3 Brewed Coffee

In general, the values for °Brix, pH and titratable acidity were similar across the production zones. The highest °Brix value corresponded to Boquete coffee samples (1.45). All brewed coffee samples showed pH values below 5.00. In terms of acidity, all samples had a slight variation, with values ranging from close to 6.00–6.35 mL of NaOH needed to reach a pH of 8.2 in 50 mL of coffee sample.

### 3.2 Sensory Evaluation

The frequency of terms used from RATA was used to identify the sensory terms perceived by the panelists across samples, and which should be included in further statistical analysis. A Cochran's *Q* test was applied to determine whether there were significant differences in the frequency of terms used. The Cochran's *Q* test results showed significant differences (*p* < 0.05) for 26 out of 28 (Tables S1 and S2) CATA descriptors. Overall, bergamot aroma and flavor were present in < 15% of the frequency responses; therefore this term was excluded from further analysis.

**FIGURE 1:**
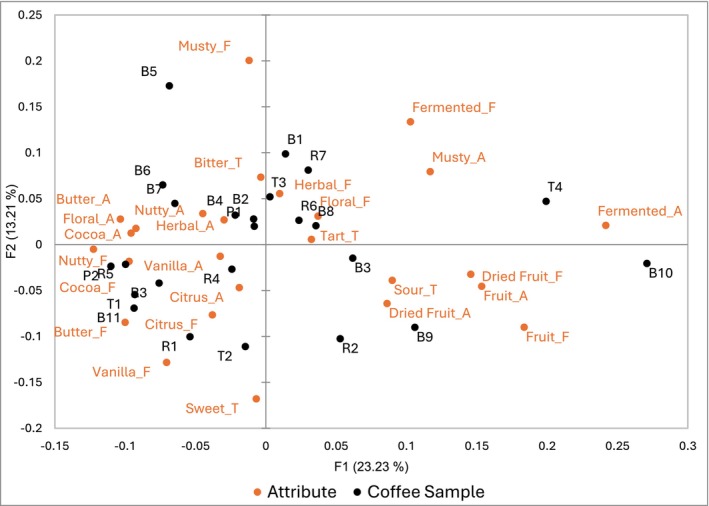
Correspondence biplot of sensory attribute descriptors (A: Aroma, F: Flavor, T: Taste) and coffee sample IDs.

The total variance observed by the Correspondence Analysis (CA) in Figure 1 was 36.44%, mainly attributed to the first two dimensions (F1: 23.23% and F2: 13.21%). The first dimension (F1) had the greatest variability, where coffee samples on the right side of the biplot were more associated with fermented, musty, and fruity attributes; whereas, on the left coffees were associated with nutty, cocoa, butter and floral descriptors. In general, musty, herbal and bitter notes contributed more to describing coffees in the positive side of the second dimension (F2), while citrus, butter, vanilla, and sweet descriptors were more linked to coffees in the lower half of the second dimension. Overall, the CA biplot showed individual coffee samples with no clear grouping based on regions, although specific differences in attributes were observed.

**FIGURE 2:**
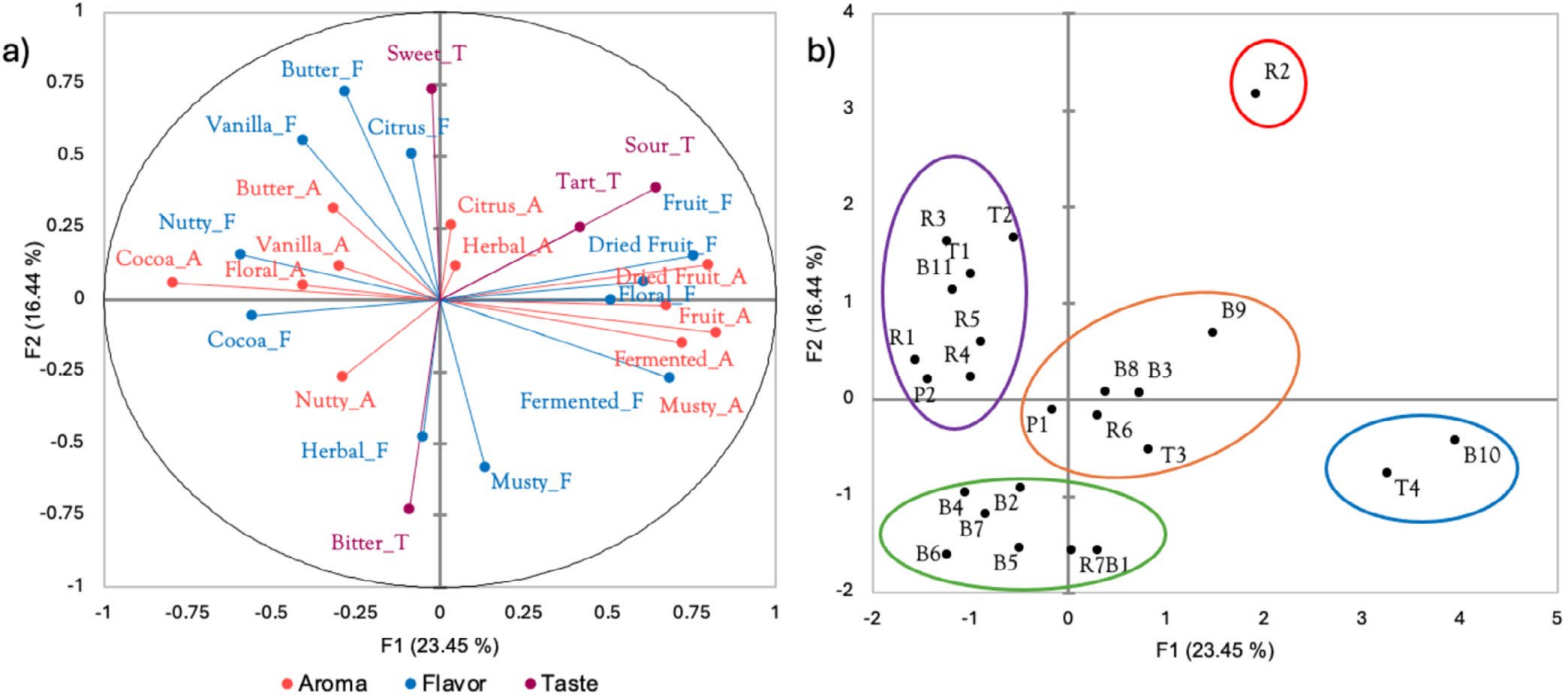
Multiple Factor Analysis (MFA) of attributes (A: Aroma, F: Flavor, T: Taste) is shown on the left (a), and clusters showing associations between individual coffee samples and attributes are shown on the biplot to the right (b). Clusters, denoted by ellipses, were determined using AHC.

MFA was used to show associations between sensory intensity data from RATA and individual coffees. The total variance for the MFA resulted in 40% across the first two dimensions (F1: 23.45% and F2: 16.44%) (Figure 2a). The aroma, flavor, and taste data were all considered different data sets. The descriptors citrus, dried fruit, vanilla, butter, cocoa, fermented and musty all had eigenvectors near to each other for aroma and flavor. Interestingly, herbal, floral, and nutty aroma and flavor eigenvectors were less associated. A similar trend is observed for sweet and bitter taste. Tart and sour taste showed a close association.

Agglomerative hierarchical clustering (AHC) was applied to the coffee data resulting in five clusters of coffee samples based on their evaluated sensorial characteristics (Figure 2b). Regional coffees were found in more than one cluster. The green cluster with six coffees from Boquete and one from Renacimiento is characterized by more intense nutty aromas, herbal and musty flavors, and a bitter taste. Two coffees were found in the blue cluster, one from Boquete and one from Tierras Altas regions, and these were characterized by more intense musty, fermented, fruit and dried fruit aromas, fermented, fruit, dried fruit and floral flavors. The orange and purple clusters showed more diversity in coffee samples, showing similar attributes association irrespective of their production zones. The orange cluster consisted of six coffees associated with citrus, nutty and herbal aromas, with some citrus flavor, including tart and sour traits in taste.

The coffees in the purple cluster presented floral, nutty, cocoa, vanilla, and butter notes in aroma. Similarly, in terms of flavor the purple cluster had predominately nutty, vanilla, cocoa and butter traits. Curiously, our analysis showed a cluster of only one coffee (R2), which was denoted to be associated predominately with a sweet taste.

**FIGURE 3:**
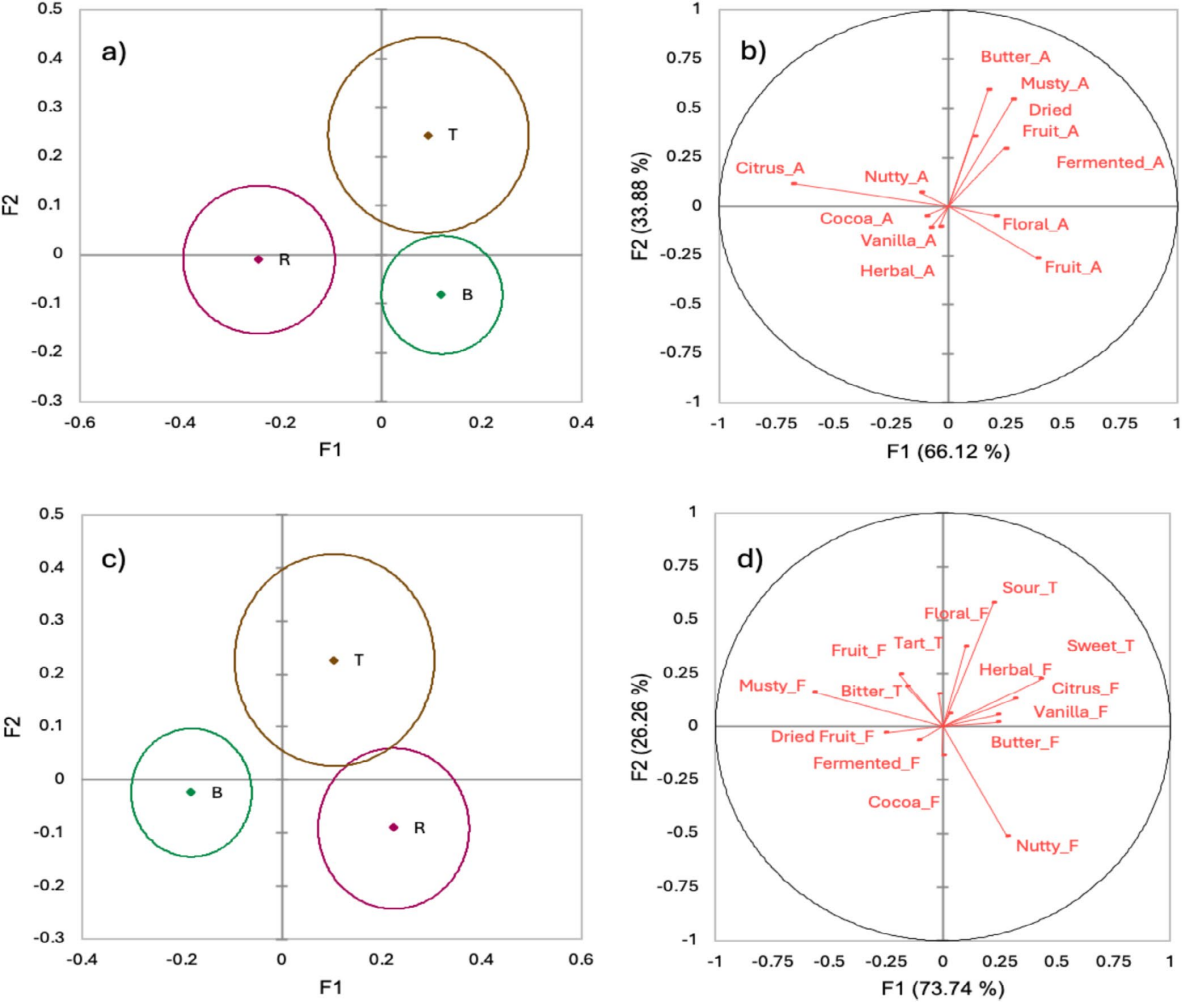
(a) Discriminant analysis centroids plot for aroma by coffee regions (B: Boquete, R: Renacimiento, T: Tierras Altas) (b) Canonical structure plot of aroma descriptors. (c) Discriminant analysis centroids plot for flavor and taste (B: Boquete, R: Renacimiento, T: Tierras Altas). (d) Canonical structure plot of flavor and taste descriptors (Flavor “F”‐Taste “T”).

Analysis of data based on production zone showed the sensory descriptors that specifically differentiate the coffees by region (Figure 3). P samples were excluded from this analysis due to a very unbalanced sample size compared to the other regions (Supporting Information). Figures to the left illustrate production zones' statistical differences with 95% confidence intervals for aroma (3a) and flavor and taste (3b). Coffee descriptors that characterized each production zone are presented in Figure 3c,d. Coffees from the Boquete region were described as having fruitier and more floral aromas, while in terms of flavor, notes of dried fruits, cocoa and fermented, along with a bitter taste were perceived in the mouth. Tierras Altas coffees were primarily characterized by floral, fruity, citrus, and herbal flavors. As for the taste, these coffees were characterized as being sour, tart, and sweet. Moreover, aroma notes of butter, dried fruits, and fermented were also perceived. Finally, coffees from Renacimiento are depicted to be more citrus, with some cocoa, herbal, and nutty aroma and flavor. Interestingly, in terms of flavor Tierras Altas and Renacimiento depicted sharing some citrus and herbal notes.

## 4 Discussion

This study aimed to determine whether terroir exists between different regions in Panama for Geisha coffee from the 2023 to 2024 crop year based on the physicochemical and sensory properties. To achieve this, conventional physicochemical analyses were conducted on green beans, roasted beans, and brewed coffee. In addition, a descriptive sensory analysis was performed using the Rate‐All‐That‐Apply method with coffee consumers, who selected aroma, flavor, and taste descriptors and rated their perceived intensities using a line scale.

Although, several terroir studies include geographical and environmental factors to define coffee terroir (Avelino et al. 2005; Decazy et al. 2003; Scholz et al. 2018; Silva et al. 2018), these approaches required multiple years of data collection, a large number of samples to ensure representativeness across regions, and complex statistical analyses. As a result, such studies are rich in data but often long‐term, and costly (Bonfante and Brillante 2022). Given these challenges, previous methodologies have combined sensory and physicochemical analyses as an alternative to characterize the quality of a product from a respective origin (Barbosa et al. 2019; Chiriboga et al. 2022; Vega et al. 2025), being a more accessible approach for further analysis. In this context, the purpose of this study was not to define Panama's coffee terroir, but rather to serve as an initial exploration of the sensory and physicochemical characteristics of Panama Geisha coffees based on coffee consumers' sensory perceptions.

Overall, the physicochemical analyses conducted on green coffee beans, roasted coffee beans, and brewed coffee showed few significant differences across regions. Moisture content and apparent density are standard quality parameters for evaluating green coffee beans, particularly in specialty coffee (ICO 2004). In this study, the moisture content reported range was from 8.82% to 9.93%. Maintaining the moisture content below 12% is important as it reduces microbial proliferation which can lead to the development of off‐odors (Scheidig et al. 2007), and bean deterioration during its storage (Gautz et al. 2008).

Coffees cultivated at altitudes above 1200 m above sea level are commercially referred to as “Strictly Hard Beans” (SHB) (J. Smith 2018), present a greater density as a typical characteristic. The coffee samples from this project were produced at high elevations (Table 1) and their apparent density was above 690 g/L; a similar value was reported by Santamaría et al. (2023), evaluating Geisha coffee from the same processing method and country of origin.

High elevation combined with lower air temperatures in coffee production systems is associated with a slower maturation of coffee cherries, resulting in denser beans (DaMatta et al. 2007). Denser beans are associated with higher quality (Alpizar and Bertrand 2005; Silva et al. 2018), many times with fruity and floral attributes present further in the cup (Decazy et al. 2003; Scholz et al. 2018; Williams et al. 2022). These quality parameters in green coffee were important to assess prior to roasting, as lower moisture content and density can lead to shorter roasting times and a risk of uneven development across samples (Gautz et al. 2008; Pereira et al. 2021). However, both parameters were consistent with specialty Geisha coffees produced in Panama. Roasted coffee samples showed expected general changes in density and color. Overall, the decrease in density is attributed to moisture loss and changes in color to Maillard reaction products such as melanoidins (Wei and Tanokura 2015), Strecker degradation, and caramelization during roasting (Condelli et al. 2022; Tarigan et al. 2022).

Roasting is known to significantly affect the development of volatile and non‐volatile compounds, producing diverse flavor coffee profiles (Giacalone et al. 2019; Koyner 2024; Tarigan et al. 2022). According to Williams et al. (2022), roasting is not considered a factor that drives terroir, because flavor and aroma precursors are already present within the green coffee beans. However, the roasting process is critical to terroir as the roasting profile can enhance or diminish the presence of those precursors and the resulting aromas and flavors (Freitas et al. 2023; Tarigan et al. 2022). Recently, Koyner (2024) demonstrated effectively how different roasting treatments could develop different floral and citrus compounds in Geisha coffees, by controlling the temperatures and time. These changes are clear examples of notable variations in coffee aroma and flavor sensory perception as a product of roasting. As roasting conditions were kept consistent, this work controlled for differences in terroir expression from roasting parameters.

Brewing method is also known to alter the composition of coffee, resulting in different flavor compositions. The temperature of the brew will greatly influence the flavor compounds, as higher temperatures tend to extract higher concentrations of compounds (Yu et al. 2021; Cai et al. 2022). The type of manual preparation within a specific temperature, such as using V60 versus French press versus drip, has also been shown to alter the volatile and nonvolatile composition of coffees, which in turn altered the sensory properties (Azizah et al. 2024). In our study, as with many other works that investigate terroir differences, we have used the same brewing process across all coffees, so the differences noted are due to the region of origin of the coffee and not the brewing method. Investigation into whether the same terroir aspects arise using a different brewing method would be of interest, as this would strengthen the terroir expression found in our study.

To minimize the effect of roasting in coffee samples, a roasting curve was developed and selected using preliminary testing (Data not shown). All samples were roasted to a light‐medium roast degree (67.60–68.85 ± 0.05) using the same temperature. The choice of this roast level allowed the flavor and aroma precursors from the beans to be highlighted, rather than those that are known to develop from a darker roast (Gonzalez‐Rios et al. 2007; Williams et al. 2022).

°Brix and pH for the brewed coffee samples were consistent with those reported by Vega et al. (2025) and Santamaría et al. (2023), considering the same coffee variety, processing method, and roast level. Similarly, the titratable acidity (TA), reported as milliliters of NaOH per 50 mL of coffee, aligned with Batali et al. (2021). The contrast between low °Brix, low pH, and high acidity is attributed to the degree of roast (Anokye‐Bempah et al. 2024; Tarigan et al. 2022), as lighter roasts thermally degrade sucrose, producing aliphatic acids (Ginz et al. 2000; Wei and Tanokura 2015) and chlorogenic acids (Anokye‐Bempah et al. 2024; Barbosa et al. 2019). Overall, the macro physicochemical parameters measured in this study appeared to be primarily influenced by the bean's composition, roast degree, and brewing method rather than by the coffee origin.

Coffee consumers were able to differentiate the coffees using 28 terms. Interestingly, the only term that was not used to differentiate coffee was “bergamot aroma and flavor.” Bergamot is a key attribute in Panama Geisha (Koyner 2024), so its presence within all samples is expected.

MFA and AHC showed five distinct clusters when coffees were investigated individually. There is some evidence for region of origin separation but only one cluster contained samples from only one region and that cluster contained only one sample. Our MFA results are very similar to those seen before where some attributes were closely related in both aroma and flavor, while others were not (Chiriboga et al. 2022). This is to be expected due to similarities in taste and aroma composition but also due to the different pathways in how sensations are perceived (retronasally vs. orthonasally) (Steen 2019; Sunarharum et al. 2014). Coffee aroma is primarily perceived through orthonasal olfaction, where volatile compounds are inhaled through the nose and retronasal olfaction is driven by the volatiles in the mouth interacting with the olfactory receptors found in the olfactory epithelium. Our physicochemical data was not shown to greatly influence the separation, but we were only looking at macro components that would correspond to tastes (Chiriboga et al. 2022; Condelli et al. 2022). Further chemical analyses, such as volatile composition analysis, are recommended to better understand the specific compounds responsible for the sensory differences.

Discriminant analysis demonstrated sensory attribute differences among the production zones. Potrerillos Arriba samples were removed due to a smaller number of samples compared to other regions. However, Boquete, Tierras Altas, and Renacimiento coffees were statistically different based on a 95% confidence interval for aroma flavor, and taste attributes (Figure 3a,c). Our study findings suggest that terroir differences do exist. From a terroir perspective, each production zone can exhibit variation in environmental conditions, or farming practices that stress the complexity and require further analysis (Williams et al. 2022). The production coffee farms considered in this study were located in different geographical points in Panama and are likely influenced by diverse microclimates. So, we can state that for harvest 2023–2024 and the coffee roasting chosen, Panama terroir does influence the Panama Geisha coffee. This work indicates that leading differences between regions B, R, and T do exist. Further research is needed to confirm these differences across different harvest seasons from the same locations and the influence of farming practices, roasting profiles, and environmental conditions.

### 4.1 Limitations of the Study

According to Giacalone et al. (2019), sensory evaluation of brewed coffee is a valuable tool to assess the quality of this beverage, and has been continuously applied in the industry or academically. The sensory method chosen will depend on the specific study objective(s). Interestingly, several studies assessing coffee terroir have considered trained panelists as well as coffee experts, using the cupping method (Alpizar and Bertrand 2005; Avelino et al. 2005; Chiriboga et al. 2022). Williams et al. (2022) mentioned that applying the cupping method is ideal to define terroir but is also limited for coffee consumer studies. Our study used coffee consumers using RATA. While it is well established that untrained panelists do perceive attributes differently from trained panelists (Yoon et al. 2023), we chose coffee consumers as they are the ultimate individuals that will purchase and enjoy these coffees. A recent method developed by the SCA, takes a more holistic approach that not only evaluates specific attributes but takes into account the origin as part of the coffee value and experience when evaluating coffees (SCA 2024b).

Our study did not delve into the reasons for the production zones' differences. Limited data bases of environmental conditions should be considered in further research.

## 5 Conclusion

This study served as an initial sensory characterization of Panama Geisha coffees from specific regions from the 2023 to 2024 crop year. Coffee consumers were able to differentiate Geisha coffee descriptors across aroma, flavor and taste attributes. Physicochemical analyses did not show significant differences across production zones. However, quality parameters such as moisture content and density of green beans were within the range of specialty coffees. Multiple Factorial Analysis (MFA) of sensory and macro‐physicochemical data did not show strong associations, suggesting additional chemical analyses to correlate with sensory differences. The roasting degree achieved contributed to determining sensory differentiations. Brewed coffees were characterized by low °Brix, low pH and high titratable acidity. Multiple Factorial Analysis (MFA) of sensory and macro‐physicochemical data did not show strong associations, suggesting additional chemical analyses to correlate with sensory differences. Finally, discriminant analysis (DA) showed significant differences in aroma, flavor and taste, across Boquete, Renacimiento, and Tierras Altas, providing evidence of terroir and regional sensory differentiation. This study shows that terroir differences do exist for Panama Geisha coffee within the different production regions, although only one roasting profile and brewing method was evaluated. Our results lay the ground work for regional profiling of Panama Geisha coffee, which can inform growers, exporters and marketers about region‐specific flavors and assist with branding strategies and enhance the value of Panama coffee within the specialty coffee market.

## Author Contributions

**Diana Batista Ledezma:** formal analysis, investigation, isualization, writing – original draft, **Camilla Sartori:** data curation, formal analysis, validation, writing – review and editing. **Elizabeth Tomasino:** conceptualization, writing – review and editing, visualization, project administration, supervision.

## Funding

This work was supported by Fulbright SENACYT.

## Ethics Statement

This study was approved by the Institutional Review Board of Oregon State University.

## Consent

Written informed consent was obtained from all study participants.

## Conflicts of Interest

The authors declare no conflicts of interest.

## Supporting information


**Figure S1:** Roasting curve model applied to coffee samples until first crack.
**Figure S2:** MFA plot combining sensory and physicochemical data (a) and biplot with clustering based on Agglomerative Hierarchical clustering (b).
**Table S1:** Summary of samples ID, production zone and range of farms elevation.
**Table S2:** Descriptors frequency Cochran's *Q* test.

## Data Availability

The data that support the findings of this study are available on request from the corresponding author. The data are not publicly available due to privacy or ethical restrictions.
