# Supplementary material for: Sensory Perception and Physicochemical Characteristics of Geisha Coffee From Different Production Zones in Panama
Source: Food Sci Nutr. 2025 Nov 25;13(12):e71278. doi: 10.1002/fsn3.71278 (PMC12645158; doi:10.1002/fsn3.71278)
Supplement: Supplementary file 1 — Figure S1: Roasting curve model applied to coffee samples until first crack. Figure S2: MFA plot combining sensory and physicochemical data (a) and biplot with clustering based on Agglomerative Hierarchical clustering (b). Table S1: Summary of samples ID, production zone and range of farms elevation. Table S2: Descriptors frequency Cochran's Q test. [file FSN3-13-e71278-s001.docx]

**Supplementary Information**


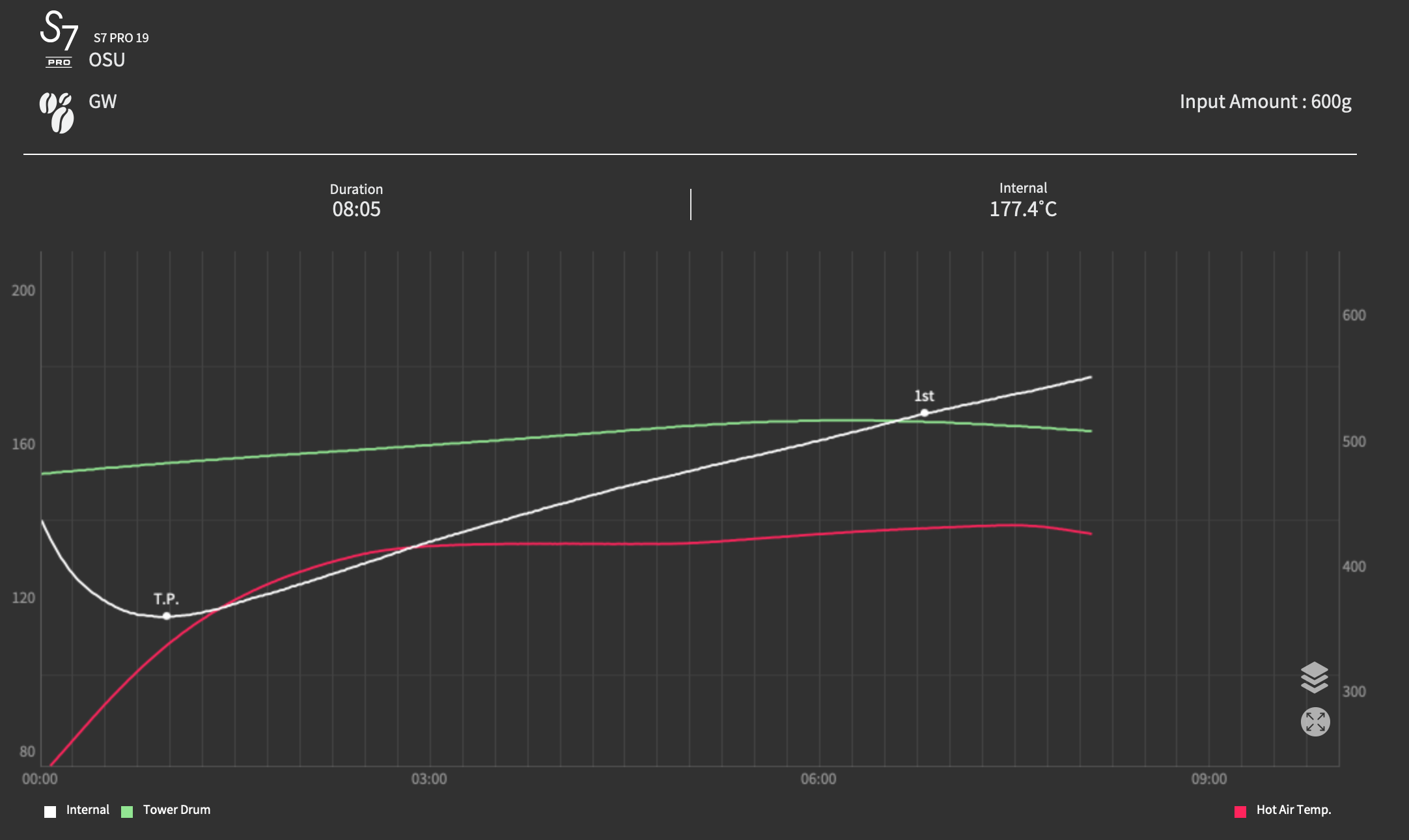


**Figure 1 S1**. Roasting curve model applied to coffee samples until first crack.


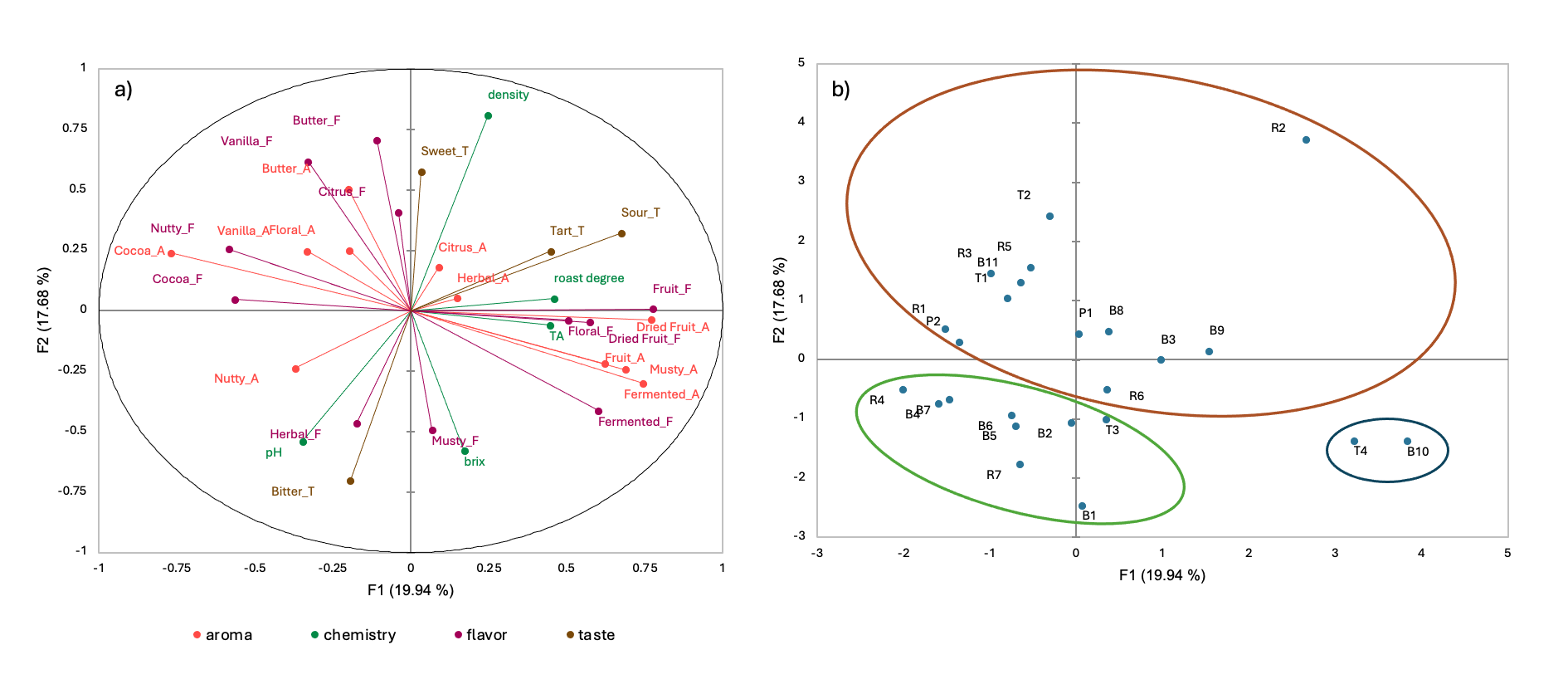


**Figure 2 S2**. MFA plot combining sensory and physicochemical data (a) and biplot with clustering based on Agglomerative Hierarchical clustering (b).

**
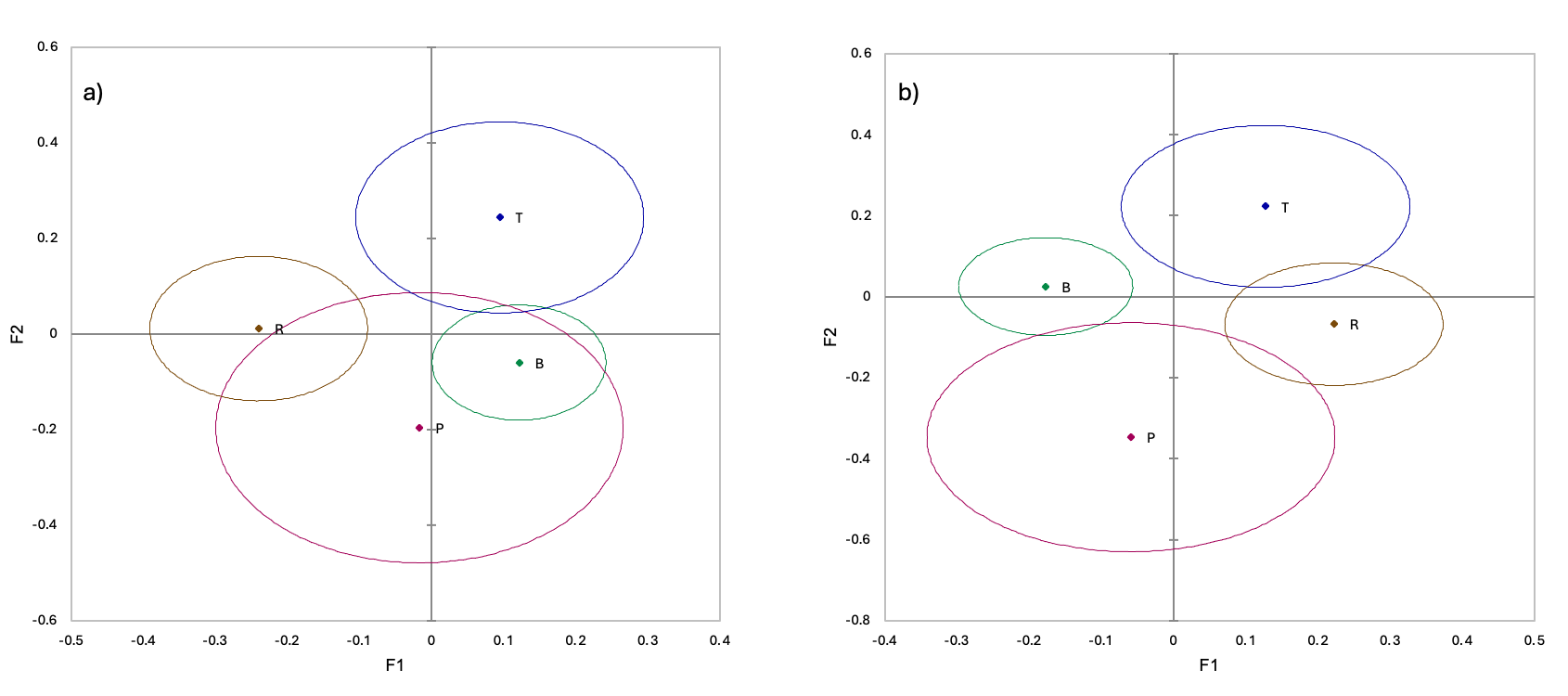
Figure 3 S 3.** Centroid plots from discriminant analysis (DA) for aroma (a) and flavor and taste (b) including Potrerillos Arriba as a production zone.

**Table 1. S1** Summary of samples ID, production zone and range of farms elevation

| **Sample ID** | **Production zone** | **Elevation range of coffee production (m.a.s.l.)** |
| --- | --- | --- |
| **B1** | Boquete | 1500-1600 |
| **B2** | Boquete | 1500 |
| **B3** | Boquete | 1650 |
| **B4** | Boquete | 1600-2000 |
| **B5** | Boquete | 1600 |
| **B6** | Boquete | 1700-1750 |
| **B7** | Boquete | 1450-1550 |
| **B8** | Boquete | 1600 |
| **B9** | Boquete | 1700 |
| **B10** | Boquete | 1800-1950 |
| **B11** | Boquete | 1700 |
| **P1** | Potrerillos Arriba | 1600 |
| **P2** | Potrerillos Arriba | 1800 |
| **T1** | Tierras Altas | 1900 |
| **T2** | Tierras Altas | 1800 |
| **T3** | Tierras Altas | 1400 |
| **T4** | Tierras Altas | 1650 |
| **R1** | Renacimiento | 1550-1740 |
| **R2** | Renacimiento | 1850 |
| **R3** | Renacimiento | - |
| **R4** | Renacimiento | 1383-1433 |
| **R5** | Renacimiento | - |
| **R6** | Renacimiento | 1400 |
| **R7** | Renacimiento | 1550 |

**Table 2. S2** Descriptors Frequency Cochran’s Q Test

| **Attribute** | **Descriptor** | **Frequencies** | **%** |
| --- | --- | --- | --- |
| Aroma | Fruit_A | 457 | 26.819 |
|  |  |  |  |
| Aroma | Floral_A | 437 | 25.646 |
|  |  |  |  |
| Aroma | **Bergamot_A** | 237 | **13.908** |
|  |  |  |  |
| Aroma | Citrus_A | 405 | 23.768 |
|  |  |  |  |
| Aroma | Butter_A | 417 | 24.472 |
|  |  |  |  |
| Aroma | Vanilla_A | 588 | 34.507 |
|  |  |  |  |
| Aroma | Cocoa_A | 743 | 43.603 |
|  |  |  |  |
| Aroma | Musty_A | 386 | 22.653 |
|  |  |  |  |
| Aroma | Nutty_A | 742 | 43.545 |
|  |  |  |  |
| Aroma | Herbal_A | 482 | 28.286 |
|  |  |  |  |
| Aroma | Fermented_A | 291 | 17.077 |
|  |  |  |  |
| Aroma | Dried Fruit_A | 416 | 24.413 |
|  |  |  |  |
| Flavor | Fruit_F | 293 | 17.195 |
|  |  |  |  |
| Flavor | Floral_F | 360 | 21.127 |
|  |  |  |  |
| Flavor | **Bergamot_F** | 248 | **14.554** |
|  |  |  |  |
| Flavor | Citrus_F | 477 | 27.993 |
|  |  |  |  |
| Flavor | Butter_F | 330 | 19.366 |
|  |  |  |  |
| Flavor | Vanilla_F | 349 | 20.481 |
|  |  |  |  |
| Flavor | Cocoa_F | 585 | 34.331 |
|  |  |  |  |
| Flavor | Musty_F | 364 | 21.362 |
|  |  |  |  |
| Flavor | Nutty_F | 602 | 35.329 |
|  |  |  |  |
| Flavor | Herbal_F | 481 | 28.228 |
|  |  |  |  |
| Flavor | Fermented_F | 289 | 16.960 |
|  |  |  |  |
| Flavor | Dried Fruit_F | 272 | 15.962 |
|  |  |  |  |
| Taste | Sweet_T | 292 | 17.136 |
|  |  |  |  |
| Taste | Sour_T | 686 | 40.258 |
|  |  |  |  |
| Taste | Bitter_T | 698 | 40.962 |
|  |  |  |  |
| Taste | Tart_T | 552 | 32.394 |

Bergamot as a descriptor in aroma and flavor had a low frequency significance < 15%.
